# Supplementary material for: Metabolic Syndrome and Colorectal Cancer Risk: Results of Propensity Score-Based Analyses in a Community-Based Cohort Study
Source: Int J Environ Res Public Health. 2020 Nov 23;17(22):8687. doi: 10.3390/ijerph17228687 (PMC7700241; doi:10.3390/ijerph17228687)

Supplementary method

This method may result in a drop-out of unmatched cases for the best matching, and thus, the number of subjects for the matching analyses were as followed; MetS: 1,038 men and 2,580 women, abnormal TG levels: 1,038 men and 2,786 women, hypertension: 1,916 men and 3,526 women, obesity: 1,412 men and 3,248 women, abnormal high-density lipoprotein cholesterol levels: 900 men and 3,884 women, abnormal fasting blood sugar: 648 men and 954 women.

Table S1. Comparison of baseline characteristics according to metabolic syndrome and abnormal triglyceride (TG) levels and differences in baseline characteristics with different propensity score-based methods in men.

Characteristics	Metabolic syndrome (N)		Standardized mean differences[a)] (N)					Abnormal TG levels (N)		Standardized mean differences [a)] (N)				
	No (1,893)	Yes (524)	Crude (2,417)	Matched (1,038)	Strata5 (2,402)	IPTW (2,417)	SMRW (2,417)	No (1,592)	Yes (825)	Crude (2,417)	Matched (1,038)	Strata5 (2,402)	IPTW (2,417)	SMRW (2,417)
Age [years, Mean (SD)]	60.31 (11.03)	57.80 (10.17)	0.237	0.032	<0.001	0.029	0.385	60.58 (11.18)	58.19 (10.15)	0.224	0.006	0.021	0.007	0.308
Physical activity [days/week, Mean (SD)]	4.09 (2.84)	3.59 (2.88)	0.175	0.019	0.019	0.019	0.267	4.01 (2.85)	3.92 (2.88)	0.243	0.037	0.020	0.018	0.331
Intake of fruits or vegetables [days/week, Mean (SD)]	5.47 (1.42)	5.24 (1.48)	0.164	0.041	0.011	0.002	0.253	5.46 (1.42)	5.36 (1.48)	0.123	0.024	0.029	0.013	0.164
Intake of beef or pork [days/week, Mean (SD)]	2.52 (1.42)	2.62 (1.31)	0.073	0.052	0.003	0.005	0.127	2.51 (1.40)	2.62 (1.38)	0.030	0.013	0.020	0.010	0.034
Alcohol consumption [N(%)]														
Non-drinkers	525 (27.7)	122 (23.3)	0.105	0.029	0.007	0.031	0.182	469 (29.46)	178 (21.58)	0.071	0.013	0.008	0.004	0.091
Moderate drinkers (<24 g/day)	640 (33.8)	183 (34.9)						560 (35.18)	263 (31.88)					
Heavy drinkers (≥24 g/day)	728 (38.5)	219 (41.8)						563 (35.36)	384 (46.55)					
Smoking status [N(%)]														
Non-smokers	381 (20.1)	114 (21.8)	0.092	0.042	0.008	0.031	0.161	338 (21.23)	157 (19.03)	0.078	0.038	0.004	0.012	0.113
Moderate smokers (<20 pack-year)	554 (29.3)	132 (25.2)						473 (29.71)	213 (25.82)					
Heavy smokers (≥20 pack-year)	958 (50.6)	278 (53.1)						781 (49.06)	455 (55.15)					
Education level [N(%)]														
Illiterate	194 (10.2)	34 (6.5)	0.211	0.049	0.016	0.029	0.337	165 (10.36)	63 (7.64)	0.118	0.017	0.011	0.014	0.168
Middle school or less	1180 (62.3)	302 (57.6)						978 (61.43)	504 (61.09)					
High school	359 (19.0)	135 (25.8)						307 (19.28)	187 (22.67)					
College or more	160 (8.5)	53 (10.1)						142 (8.92)	71 (8.61)					
Residential area [N(%)]														
Sancheong-gun	1046 (55.3)	247 (47.1)	0.312	0.027	0.068	0.011	0.448	867 (54.46)	426 (51.64)	0.141	0.030	0.005	0.012	0.185
Changwon-si	410 (21.7)	91 (17.4)						340 (21.36)	161 (19.52)					
Chooncheon-si	130 (6.9)	44 (8.4)						109 (6.85)	65 (7.88)					
Choongjoo-si	185 (9.8)	104 (19.8)						167 (10.49)	122 (14.79)					
Haman-gun	122 (6.4)	38 (7.3)						109 (6.85)	51 (6.18)					

SD, standard deviation; Crude, whole dataset; Matched, 1:1 matched dataset; Strata5, dataset stratified with 5 strata; IPTW, inverse probability-of-treatment weighted dataset; SMRW, standardized mortality ratio weighted dataset. [a)] The values less than 0.1 were considered negligible differences.

Table S2. Comparison of baseline characteristics according to metabolic syndrome and abnormal triglyceride (TG) levels and differences in baseline characteristics with different propensity score-based methods in women.

Characteristics	Metabolic syndrome (N)		Standardized mean differences[a)] (N)					Abnormal TG levels (N)		Standardized mean differences [a)] (N)				
	No (3,271)	Yes (1,397)	Crude (4,568)	Matched (2,580)	Strata5 (4,558)	IPTW (4,568)	SMRW (4,568)	No (3,175)	Yes (1,393)	Crude (4,568)	Matched (2,786)	Strata5 (4,554)	IPTW (4,568)	SMRW (4,568)
Age [years, Mean (SD)]	59.38 (11.72)	61.59 (9.54)	0.206	0.016	0.039	0.036	0.428	59.09 (11.74)	62.10 (9.47)	0.282	0.008	0.021	0.021	0.472
Physical activity [days/week, Mean (SD)]	3.13 (2.96)	2.65 (2.92)	0.164	0.02	0.001	0.006	0.199	3.08 (2.96)	2.78 (2.94)	0.102	0.042	0.017	0.003	0.121
Intake of fruits or vegetables [days/week, Mean (SD)]	5.55 (1.43)	5.37 (1.53)	0.127	0.004	0.002	0.004	0.173	5.51 (1.45)	5.48 (1.50)	0.021	0.011	0.011	0.008	0.028
Intake of beef or pork [days/week, Mean (SD)]	1.77 (1.34)	1.67 (1.31)	0.071	0.029	0.029	0.007	0.166	1.81 (1.34)	1.60 (1.31)	0.158	0.021	0.006	0.005	0.263
Alcohol consumption [N(%)]														
Non-drinkers	2560 (78.3)	1083 (83.5)	0.137	0.027	0.011	0.007	0.255	2473 (77.9)	1170 (84.0)	0.156	0.017	0.008	0.009	0.261
Moderate drinkers (<24 g/day)	615 (18.8)	190 (14.6)						612 (19.3)	193 (13.9)					
Heavy drinkers (≥24 g/day)	96 (2.9)	24 (1.9)						90 (2.8)	30 (2.2)					
Smoking status [N(%)]														
Non-smokers	3028 (92.6)	1210 (93.3)	0.034	0.016	0.011	0.008	0.056	2958 (93.2)	1280 (91.9)	0.059	0.037	0.016	0.004	0.084
Moderate smokers (<20 pack-year)	197 (6.0)	68 (5.2)						178 (5.6)	87 (6.2)					
Heavy smokers (≥20 pack-year)	46 (1.4)	19 (1.5)						39 (1.2)	26 (1.9)					
Education level [N(%)]														
Illiterate	1040 (31.8)	429 (33.1)	0.263	0.056	0.111	0.023	0.534	993 (31.3)	476 (34.2)	0.246	0.034	0.052	0.012	0.441
Middle school or less	1757 (53.7)	774 (59.7)						1716 (54.0)	815 (58.5)					
High school	356 (10.9)	85 (6.6)						354 (11.1)	87 (6.2)					
College or more	118 (3.6)	9 (0.7)						112 (3.5)	15 (1.1)					
Residential area [N(%)]														
Sancheong-gun	1830 (55.9)	571 (44.0)	0.348	0.018	0.078	0.01	0.459	1724 (54.3)	677 (48.6)	0.159	0.031	0.057	0.007	0.214
Changwon-si	653 (20.0)	227 (17.5)						619 (19.5)	261 (18.7)					
Chooncheon-si	304 (9.3)	137 (10.6)						291 (9.2)	150 (10.8)					
Choongjoo-si	311 (9.5)	258 (19.9)						351 (11.1)	218 (15.6)					
Haman-gun	173 (5.3)	104 (8.0)						190 (6.0)	87 (6.2)					

SD, standard deviation; Crude, whole dataset; Matched, 1:1 matched dataset; Strata5, dataset stratified with 5 strata; IPTW, inverse probability-of-treatment weighted dataset; SMRW, standardized mortality ratio weighted dataset. [a)] The values less than 0.1 were considered negligible differences.

Table S3. Associations between (a) bool pressure, (b) obesity, (c) HDL, (d) FBS and Colorectal Cancer risk.

	Total				Men				Women			
**Methods**	**Cases (N)**	**Controls (N)**	**HR (95% CI)**	***P*-value**	**Cases (N)**	**Controls (N)**	**HR (95% CI)**	***P*-value**	**Cases (N)**	**Controls (N)**	**HR (95% CI)**	***P*-value**
**(a) BP (SBP≥130 mmHg and DBP ≥85 mmHg)**												
**General Cox hazard regression**												
Unadjusted	111	6874	1.36 (0.92, 2)	0.12	57	2,360	1.24 (0.72, 2.12)	0.435	54	4,514	1.45 (0.83, 2.54)	0.193
Multivariable[a)]	111	6874	1.1 (0.74, 1.64)	0.628	57	2,360	1.02 (0.58, 1.78)	0.944	54	4,514	1.17 (0.66, 2.08)	0.589
**PS-based Cox hazard regression**												
Matched for PS	80	5424	0.99 (0.64, 1.54)	0.969	42	1,874	1.00 (0.54, 1.82)	0.987	42	3,484	1.33 (0.72, 2.45)	0.362
Stratification into 5 strata by PS	111	6871	1.11 (0.77, 1.6)	0.567	57	2,350	1.07 (0.64, 1.79)	0.801	54	4,474	1.11 (0.66, 1.87)	0.694
Regression adjusted with PS												
as a continuous term	111	6874	1.07 (0.72, 1.6)	0.747	57	2,360	1.02 (0.58, 1.78)	0.953	54	4,514	1.11 (0.62, 1.99)	0.719
as a quintile term	111	6874	1.11 (0.75, 1.65)	0.605	57	2,360	1.05 (0.6, 1.82)	0.874	54	4,514	1.14 (0.64, 2.03)	0.65
Weighted models												
IPTW model	111	6874	1.12 (0.86, 1.45)	0.419	57	2,360	1.07 (0.74, 1.55)	0.738	54	4,514	1.16 (0.79, 1.70)	0.444
SMRW model	111	6874	1.36 (1.03, 1.79)	0.03	57	2,360	1.23 (0.84, 1.81)	0.294	54	4,514	1.46 (0.98, 2.16)	0.062
**(b) Obesity (BMI (kg/m^2) ≥ 25)**												
**General Cox hazard regression**												
Unadjusted	111	6874	0.96 (0.65, 1.42)	0.832	57	2,360	0.98 (0.56, 1.74)	0.955	54	4,514	1.05 (0.61, 1.81)	0.866
Multivariable[a)]	111	6874	1.18 (0.78, 1.77)	0.436	57	2,360	1.19 (0.66, 2.14)	0.573	54	4,514	1.19 (0.68, 2.08)	0.552
**PS-based Cox's hazard regression**												
Matched for PS	69	4695	1.22 (0.76, 1.96)	0.414	37	1,375	0.85 (0.44, 1.62)	0.615	43	3.205	0.95 (0.52, 1.73)	0.873
Stratification into 5 strata by PS	110	6861	1.07 (0.72, 1.6)	0.735	56	2,352	1.09 (0.61, 1.93)	0.77	54	4,510	1.15 (0.66, 2.00)	0.62
Regression adjusted with PS												
as a continuous term	111	6874	1.09 (0.73, 1.63)	0.673	57	2,360	1.17 (0.65, 2.11)	0.604	54	4,514	1.15 (0.66, 2.00)	0.635
as a quintile term	111	6874	1.07 (0.72, 1.6)	0.726	57	2,360	1.18 (0.66, 2.13)	0.573	54	4,514	1.17 (0.67, 2.04)	0.59
Weighted models												
IPTW model	111	6874	1.14 (0.88, 1.47)	0.336	57	2,360	1.26 (0.89, 1.79)	0.2	54	4,514	1.15 (0.79, 1.67)	0.469
SMRW model	111	6874	0.94 (0.67, 1.31)	0.704	57	2,360	0.98 (0.6, 1.61)	0.946	54	4,514	1.03 (0.65, 1.62)	0.909
**(c) HDL cholesterol (<40 mg/dL for men; <50 mg/dL for women)**												
**General Cox hazard regression**												
Unadjusted	111	6874	0.61 (0.4, 0.92)	0.019	57	2,360	0.24 (0.08, 0.77)	0.016	54	4,514	1.12 (0.65, 1.90)	0.687
Multivariable[a)]	111	6874	0.71 (0.45, 1.11)	0.129	57	2,360	0.27 (0.08, 0.88)	0.029	54	4,514	0.92 (0.53, 1.60)	0.767
**PS-based Cox hazard regression**												
Matched for PS	57	4721	0.68 (0.4, 1.15)	0.153	12	888	0.33 (0.09, 1.22)	0.097	44	3,840	0.84 (0.46, 1.52)	0.559
Stratification into 5 strata by PS	109	6804	0.74 (0.48, 1.13)	0.157	57	2,350	0.27 (0.08, 0.85)	0.026	54	4,506	0.97 (0.58, 1.62)	0.899
Regression adjusted with PS												
as a continuous term	111	6874	0.71 (0.46, 1.1)	0.127	57	2,360	0.28 (0.09, 0.92)	0.036	54	4,514	0.96 (0.56, 1.66)	0.883
as a quintile term	111	6874	0.71 (0.45, 1.11)	0.133	57	2,360	0.27 (0.08, 0.89)	0.031	54	4,514	0.97 (0.56, 1.67)	0.901
Weighted models												
IPTW model	111	6874	0.63 (0.47, 0.84)	0.002	57	2,360	0.41 (0.26, 0.64)	<0.001	54	4,514	0.96 (0.65, 1.40)	0.813
SMRW model	111	6874	0.53 (0.37, 0.76)	0.001	57	2,360	0.22 (0.07, 0.70)	0.01	54	4,514	1.12 (0.74, 1.70)	0.584

**(d) FBS (≥ 100 mg/dL)**												
**General Cox's hazard regression**												
Unadjusted	111	6874	1.42 (0.84, 2.41)	0.196	57	2,360	1.69 (0.88, 3.27)	0.117	54	4,514	0.95 (0.38, 2.39)	0.913
Multivariable[a)]	111	6874	1.24 (0.73, 2.12)	0.425	57	2,360	1.74 (0.90, 3.40)	0.103	54	4,514	0.74 (0.29, 1.88)	0.527
**PS-based Cox's hazard regression**												
Matched for PS	32	1570	1.07 (0.53, 2.14)	0.854	14	634	3.99 (1.11, 14.32)	0.034	10	944	1.06 (0.31, 3.67)	0.925
Stratification into 5 strata by PS	111	6857	1.24 (0.74, 2.1)	0.417	55	2,290	1.82 (0.93, 3.54)	0.078	53	4,461	0.76 (0.31, 1.89)	0.554
Regression adjusted with PS												
as a continuous term	111	6874	1.22 (0.72, 2.08)	0.466	57	2,360	1.69 (0.87, 3.29)	0.121	54	4,514	0.74 (0.29, 1.89)	0.534
as a quintile term	111	6874	1.24 (0.73, 2.12)	0.43	57	2,360	1.76 (0.90, 3.42)	0.098	54	4,514	0.77 (0.30, 1.95)	0.58
Weighted models												
IPTW model	111	6874	1.15 (0.89, 1.5)	0.286	57	2,360	1.58 (1.11, 2.25)	0.011	54	4,514	0.82 (0.55, 1.22)	0.319
SMRW model	111	6874	1.63 (0.99, 2.67)	0.053	57	2,360	1.64 (0.90, 2.98)	0.106	54	4,514	1.19 (0.49, 2.86)	0.702

HR, hazard ratio; CI, confidence interval; PS, propensity score; IPTW, inverse probability-of-treatment weighted; SMRW, standardized mortality ratio weighted. [a)] Adjusted by age, sex (in case of total) education, smoking status, alcohol consumption, physical activity, frequency of intake of fruits or vegetables, frequency of intake of red meats, and residential area.

Supplementary Figure S1. Comparison of the standardized mean differences by covariates with different propensity score-based methods according to (a) metabolic syndrome and (b) triglyceride level in men. Crude, whole dataset; Matched, 1:1 matched dataset; Strata5, dataset stratified into 5 strata; IPTW, inverse-probability-of-treatment-weighted dataset; SMRW, standardized mortality ratio-weighted dataset. This plot shows the standardized mean differences between study subjects who have metabolic syndrome and those who do not; a value > 0.1 indicates imbalance of a covariate.

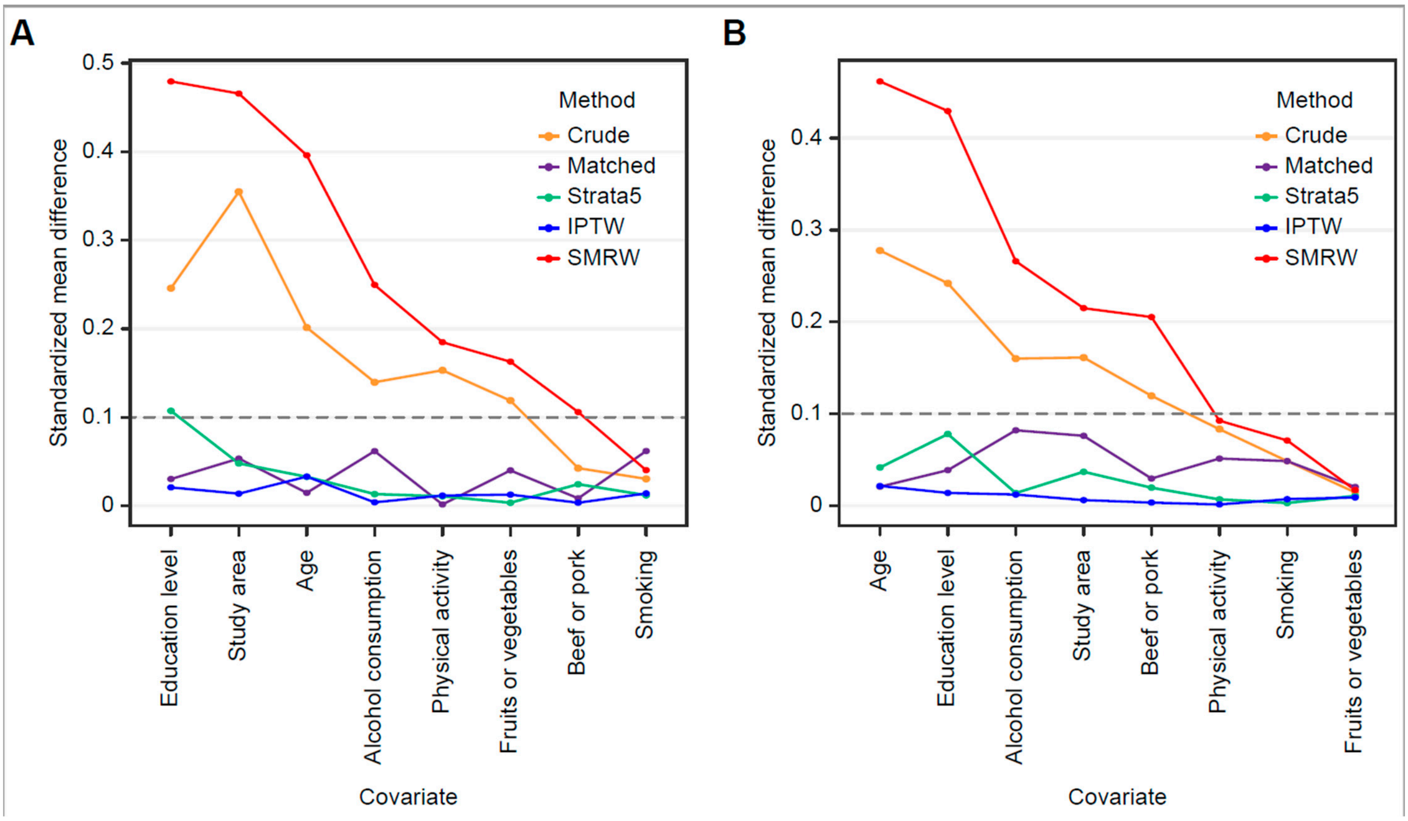

Supplementary Figure S2. Comparison of the standardized mean differences by covariates with different propensity score-based methods according to (a) metabolic syndrome and (b) triglyceride level in women. Crude, whole dataset; Matched, 1:1 matched dataset; Strata5, dataset stratified into 5 strata; IPTW, inverse-probability-of-treatment-weighted dataset; SMRW, standardized mortality ratio-weighted dataset. This plot shows the standardized mean differences between study subjects who have metabolic syndrome and those who do not; a value > 0.1 indicates imbalance of a covariate.

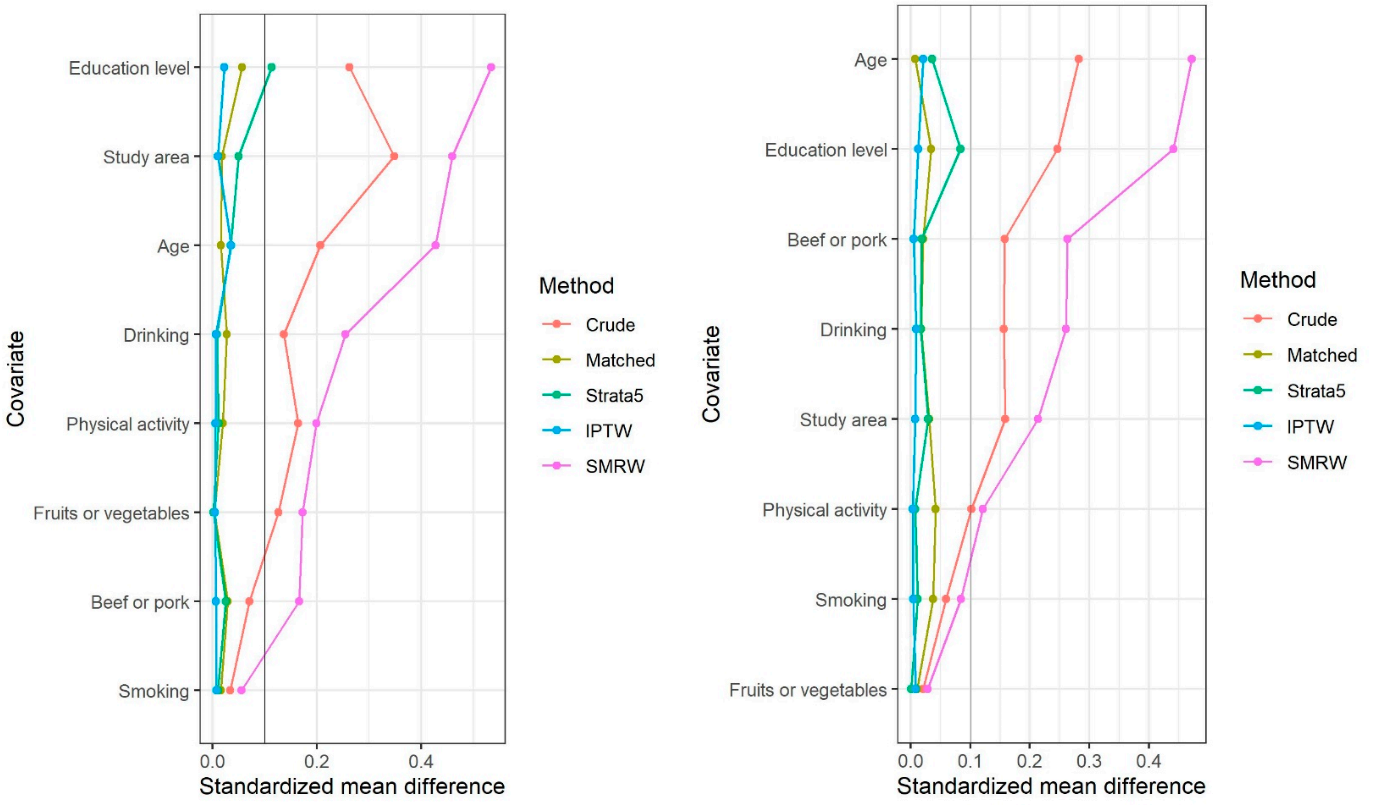

Supplement: Supplementary file 1 [file ijerph-17-08687-s001.pdf]
